# Identifying potential threats to soil biodiversity

**DOI:** 10.7717/peerj.9271

**Published:** 2020-06-12

**Authors:** Mark Tibbett, Tandra D. Fraser, Sarah Duddigan

**Affiliations:** Department of Sustainable Land Management and Soil Research Centre, School of Agriculture Policy and Development, University of Reading, Reading, Berkshire, United Kingdom

**Keywords:** Soil ecology, Biodiversity, Soil fauna, Soil microbiology, Functional redundancy, Mycorrhiza, Molecular ecology, Soil communities, Soil functions, Ecoystem services

## Abstract

A decline in soil biodiversity is generally considered to be the reduction of forms of life living in soils, both in terms of quantity and variety. Where soil biodiversity decline occurs, it can significantly affect the soils’ ability to function, respond to perturbations and recover from a disturbance. Several soil threats have been identified as having negative effects on soil biodiversity, including human intensive exploitation, land-use change and soil organic matter decline. In this review we consider what we mean by soil biodiversity, and why it is important to monitor. After a thorough review of the literature identified on a Web of Science search concerning threats to soil biodiversity (topic search: threat* “soil biodiversity”), we compiled a table of biodiversity threats considered in each paper including climate change, land use change, intensive human exploitation, decline in soil health or plastic; followed by detailed listings of threats studied. This we compared to a previously published expert assessment of threats to soil biodiversity. In addition, we identified emerging threats, particularly microplastics, in the 10 years following these knowledge based rankings. We found that many soil biodiversity studies do not focus on biodiversity sensu stricto, rather these studies examined either changes in abundance and/or diversity of individual groups of soil biota, instead of soil biodiversity as a whole, encompassing all levels of the soil food web. This highlights the complexity of soil biodiversity which is often impractical to assess in all but the largest studies. Published global scientific activity was only partially related to the threats identified by the expert panel assessment. The number of threats and the priority given to the threats (by number of publications) were quite different, indicating a disparity between research actions versus perceived threats. The lack of research effort in key areas of high priority in the threats to soil biodiversity are a concerning finding and requires some consideration and debate in the research community.

## Introduction

**Figure 1:**
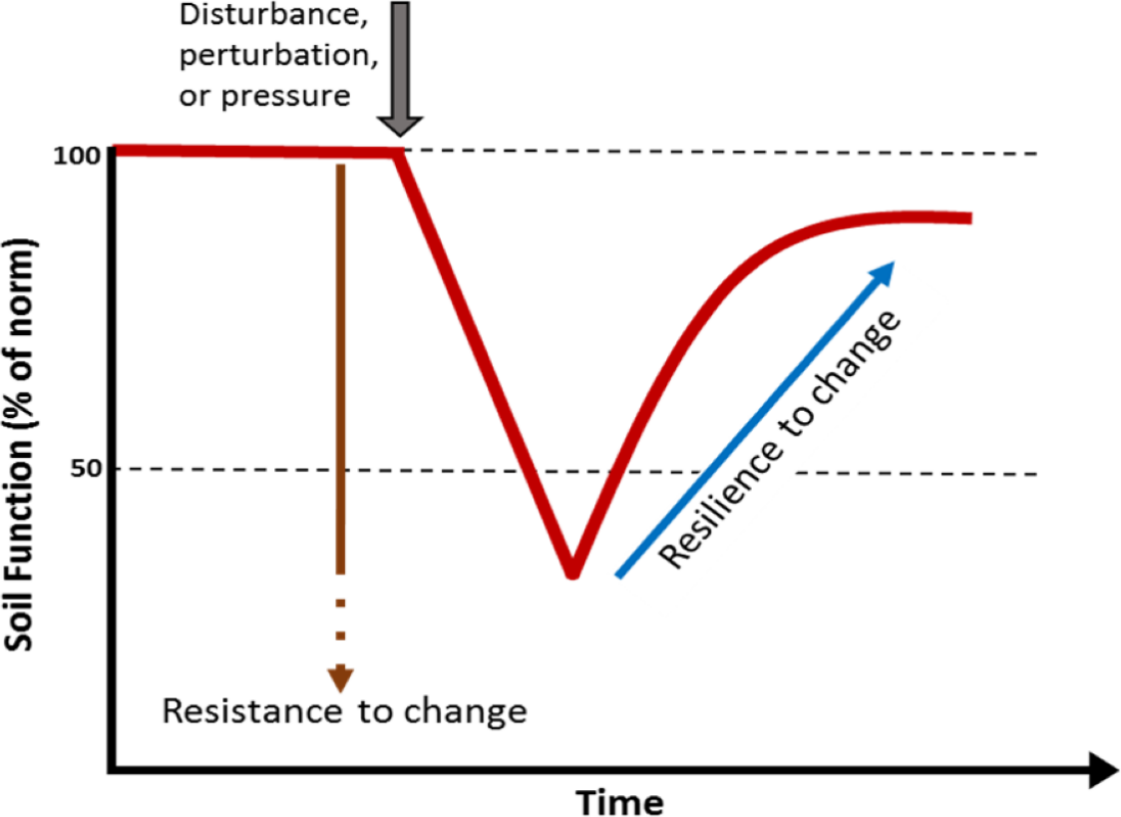
Simple model showing the effect of a perturbation on the resistance and resilience of a soil biological function or property. Higher biodiversity is thought to correspond to high resistance and resilience. A loss of biodiversity is thought lead to a soil with lower resistance to a perturbation and lower capacity to recover.

Soils are a globally important reservoir of biodiversity, hosting at least one quarter of all living organisms on the planet (Decaëns et al., 2006). Soil provides a variety of functions and services supporting life on the planet including: generation of biomass; biogeochemical cycling; and regulation of water movement, climate and pollution (Adhikari & Hartemink, 2016; Blouin et al., 2013; Chen et al., 2020). However, the ability of soils to provide these services is highly dependent on their biodiversity (Bardgett & Van Der Putten, 2014; Pulleman et al., 2012), to such an extent that soil biodiversity is often recognised as a cornerstone for soil security (McBratney, Field & Koch, 2014). The soil biota has its own unique capacity to resist events that cause disturbance or change and a certain capacity to recover from these perturbations. The capacity to recover from change is considered a key attribute of biodiversity. Soils with higher biodiversity are thought to have an innate resistance and resilience to change (Fig. 1). A loss of biodiversity can lead to a soil with lower resistance to a perturbation and reduced capacity to recover (Allison & Martiny, 2008; Downing et al., 2012).

The decline in soil biodiversity is generally considered to be the reduction of forms of life living in soils, both in terms of quantity and variety (Jones et al., 2005). Wherever soil biodiversity decline occurs it can significantly affect the soils’ ability to function normally, respond to perturbations and the capacity to recover. Several soil threats have been identified as having negative effects on soil biodiversity, including human intensive exploitation, land-use change and soil organic matter decline (Gardi, Jeffery & Saltelli, 2013).

Predictive maps describing the state of soil biodiversity, both in general and geographical terms, exist in atlases of soil biodiversity (Jeffery et al., 2010; Orgiazzi, Bardgett & Barrios, 2016) and at some local levels it is clear that soil biodiversity is in decline, at least for some taxa. In addition, maps for particular organisms such as bacteria (Delgado-Baquerizo et al., 2018), fungi (Tedersoo et al., 2014), nematodes (van den Hoogen et al., 2019) and earthworms (Phillips et al., 2019) have also been constructed to aid in our understanding of their global distributions.

With the use of expert assessment, the authors of these atlases have highlighted 12 potential threats to soil biodiversity, which have subsequently been used to estimate the magnitude and map potential threats to soil biodiversity (Jeffery & Gardi, 2010; Orgiazzi, Bardgett & Barrios, 2016). These were, in order of weighting by experts in Jeffery & Gardi (2010): (1) Human intensive exploitation; (2) Soil organic matter decline; (3) Habitat disruption; (4) Soil sealing; (5) Soil pollution; (6) Land use change; (7) Soil compaction; (8) Soil erosion; (9) Habitat fragmentation; (10) Climate change; (11) Invasive species; and (12) GMO pollution. Orgiazzi, Bardgett & Barrios (2016) combined information from ranked soil threats with ad-hoc proxies, to determine the spatial distribution of threats on soil microorganisms, soil fauna, and functions for 27 countries across Europe (although habitat disruption was replaced with soil salinization). A large proportion of these soils (more than 40% in 14 of the countries) were categorized as having moderately high to high potential threats to biodiversity and function, with arable soil most at risk.

After discussing what we mean by soil biodiversity, and why it is important to monitor, this review will discuss whether emphasis in the literature surrounding threats to soil biodiversity reflects the results of this expert assessment. In addition, we will identify whether there have been any emerging threats in the 10 years following these knowledge based rankings.

## Methods

Using Web of Science a basic literature search was conducted with the ‘topic’ field. A topic field search will examine the title, abstract and keywords of every record. This review used the search term: threat* “soil biodiversity” on 27th November 2019, which returned 72 results. Note the key and specific term soil biodiversity was used rather than the generic term soil biology, nonetheless, some of the references returned (particularly experimental papers) often addresses the effects of certain threats on a single group of organisms or species. Despite this, these studies were still included. The use of threat* will return records that use the term threat, threats or threatened with soil biodiversity in the title, abstract or keywords. We recognise that papers discussing negative effects may not all be returned using the term “threat”. However, when the work considers sufficient damage or harm is likely to be caused to soil biodiversity then the term “threat” will almost inevitably be used. After datasets, duplicates, and references that were not relevant were omitted (e.g., studies on the human gut microbiome), 46 papers were left for review. Each reference was studied in detail, and the potential threats to soil biodiversity that each paper identified was compiled into a compressive table describing the type of study (experimental, review or meta-analysis) and the threat(s) to biodiversity considered in the work.

## Description of soil biodiversity

Biodiversity itself is a relatively recent concept first used in 1988 (Wilson & Peter, 1998) and has been defined in different ways, but most simply put is *the variety of life*. Soil biodiversity is generally defined as the variability of living organisms in soil and the ecological complexes of which they are part; this includes diversity within species, between species and of ecosystems (UNEP, 1992). For the purposes of soils, and its complexity of habitats a more detailed definition should incorporate the variety and variability of living organisms along with the ecological complexes in which they occur. This may encompass the following definitions (after Jensen, Torn & Harte, 1990) as: (1) **ecosystem diversity** considering the variety of habitats that are in the soil, (2) **species diversity** where the variety and abundance of different types of organisms inhabiting a soil (akin to taxonomic diversity), (3) **genetic diversity** as the combination of different genes found within a population of a single species, and the pattern of variation found within different populations of the same species (can also be assessed across the whole community of organisms), (4) **phenotypic diversity** based on any and/or all of the morphological, biochemical or physiological aspects of the organism in the soil and is a result of genes and environmental factors, and (5) **functional diversity** as the variety of functions performed by the soil biota (e.g., such as nitrification, litter comminution and carbon turnover). On review of the 46 papers returned in our Web of Science search, only species, functional and ecosystem diversity were discussed. With the vast majority of the literature focussed on species diversity, although functional diversity in the context of ecosystems was also addressed in c. 20% of the papers.

## Species diversity

**Figure 2:**
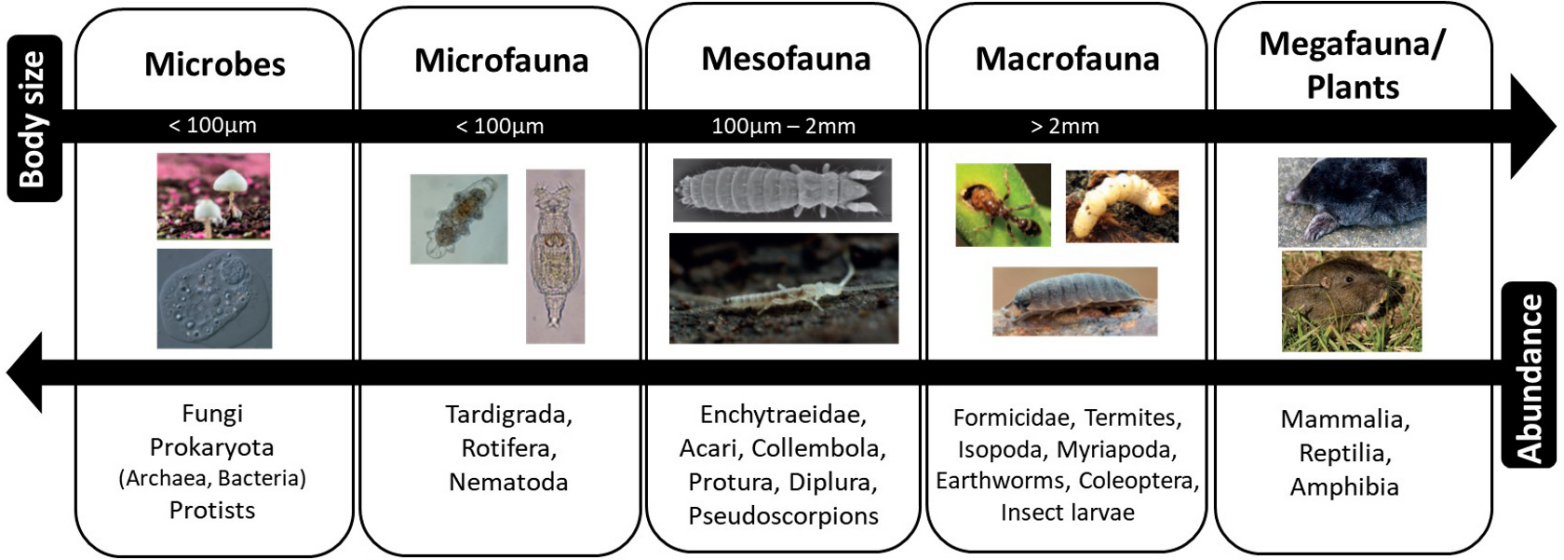
Size classification of soil organisms. As body size increases, abundance decreases. Photos from Global Soil Biodiversity Atlas (Orgiazzi et al. 2016); Credit: B Jakabek, Y Eglit, M Shaw, H Segers, L Galli, A Murray, RR Castro Solar, T Tsunoda, S Franzenburg, D Hope, C Abbe.

At its simplest, the vast biodiversity of the soil can be divided into five major groups (Fig. 2). These are: (i) microbes and (ii) microfauna with body widths of less than 100 µm; (iii) mesofauna with body widths between 100 µm and 2 mm; (iv) macrofauna and (v) megafauna that are larger than 2 mm (Swift, Heal & Anderson, 1979; Wurst et al., 2012). While the size boundaries for classification into micro, meso, and macro are universally agreed, published groupings and classification can vary as some taxa cross size boundaries, and may be interpreted in terms of body width or length (Coleman, Callaham & Crossley, 2018; Swift, Heal & Anderson, 1979).

The microbes are the smallest group in physical dimension yet the most abundant and, despite their size, may comprise the largest biomass. The microbial community is the most diverse group of organisms, not only in the soil but arguably on the planet. The major taxa comprise bacteria, archaea, fungi, protists and viruses (Fierer et al., 2007). The biodiversity of soil bacterial communities alone is enormous where one gram of soil may contain anything from ten thousand to ten million taxa (Gans, Wolinsky & Dunbar, 2005; Torsvik, Ovres & Thingstad, 2002; Tringe et al., 2005). The diversity and roles of microbial symbionts (e.g., N-fixers and mycorrhiza) should not be underestimated as these are key components of many terrestrial ecosystems (Chaia, Wall & Huss-Danell, 2010; Hayat, Ali & Amara, 2010; Kariman, Barker & Tibbett, 2018). The protists are the most diverse group and are single celled eukaryotes. They are primary and secondary consumers, predominantly feeding on bacteria and fungi with some saprophytic taxa also present in soil, and therefore have an important role in nutrient cycles through mineralization of carbon, nitrogen, phosphorus and silica (Wurst et al., 2012; Nielsen, 2019; Oliverio et al., 2020). In addition, protists influence soil food webs and processes through their actions as primary producers, predators of other eukaryotes, decomposers and parasites or pathogens to larger flora and fauna (Oliverio et al., 2020) The microfauna consist of tiny soil animals (<0.1 mm) that are dominated by three main groups Tardigrada, Nematoda and Rotifera. They usually require water films or water filled pores to move around the soil and feed and are therefore referred to as aquatic organisms (Nielsen, 2019; Wurst et al., 2012; Coleman, Callaham & Crossley, 2018). The multicellular nematodes, which are small round worms, are the most abundant animals on earth, accounting for around 80% of all animals on land (Van den Hoogen et al., 2019). Nematodes have a wide range of feeding strategies and may be microphagous as well as plant parasites (Nielsen, 2019). The Rotifera, which are also multicelled, primarily feed on bacteria and algae (Wurst et al., 2012). The mesofauna include arthropods, such as mites, collembola (springtails) and enchytraeids and many other groups (Jeffery et al., 2010). They tend to occupy air-filled pores in soil and litter and feed on the microbes and microfauna as well as plants and algae. The macrofauna includes snails, slugs, earthworms, ants, termites, millipedes, woodlice and larger megafauna such as moles, potoroos, wombats badgers and rabbits. Burrowing animals such as earthworms, ants and millipedes create their own living space by burrowing into the soil and as such can alter the soil. These groups are sometimes referred to as “ecosystem engineers” (Jones, Lawton & Shachak, 1996; Lavelle & Spain, 2001).

There are several commonly used ways of assessing species diversity that are based on calculated metrics or indices (Magurran, 2004). The most simplistic measure is *species richness* which is simply the number of species present in the soil, or more accurately in the sample(s) of soil taken. The problem with this metric is that common species are found with little sampling effort and more with greater sampling effort. Therefore, this is no longer recognised as a comprehensive measure of biodiversity. In order to avoid this potential misinterpretation of diversity, numerous indices have been developed that suit a variety of environmental and community circumstances (see Magurran (2004) for a comprehensive discussion). Two biodiversity metrics are used commonly to calculate relative abundance from proportions (*p*_*i*_) of each species (*i*) within the total number of individuals. Simpson’s Index:

(1) \begin{eqnarray*}D=1/\sum p_{i}^{2}\end{eqnarray*}

where *D* equals diversity. For any number of species in a sample (*S*), the value of *D* can range from 1 to *S*. As *D* increases diversity decreases so this index is usually expressed as 1–D or 1/D. In contrast, the Shannon-Weaver Index (*H*) is a logarithmic measure of diversity:

(2) \begin{eqnarray*}H=-\sum {p}_{i}{\mathrm{log}}_{\mathrm{e}}{p}_{i}\end{eqnarray*}

The higher *H*, the greater the diversity. Because H is roughly proportional to the logarithm of the number of species, it is sometimes preferable to present data as e^*H*^, which is proportional to the actual number of species.

The scale at which biodiversity is considered is also important, and the type of biodiversity measured is dependent on whether comparisons are made with or between soils (habitats). *Alpha-diversity*, or within-habitat diversity, refers to a group of organisms interacting and competing for the same resources or sharing the same environment or soil. This is measured as the number of species within a given area. *Beta-diversity*, or between-habitat diversity, refers to the response of organisms to spatial heterogeneity. High beta-diversity implies low similarity between species composition of different soils or habitats. It is usually expressed in terms of similarity index between communities between different habitats in same geographical area. *Gamma diversity*, or landscape diversity, refers to the total biodiversity over a large area or region. It is the total of α and β diversity.

Loss of species diversity can often be hard to quantify especially at smaller scales as it is likely that some species could be extinct before they are recorded or described (Jeffery & Gardi, 2010). It is also difficult to establish whether unobserved microbes are extinct due to the vast array of microhabitats they may occupy (discussed later). Moreover, in the context of ecosystem services, it is important to determine whether all species are equally important in carrying out certain functions, or whether some contribute more than others (Blouin et al., 2013). Rare species can drive key processes, such as nutrient cycling, greenhouse gas emissions and pollutant degradation (Jousset et al., 2017)

## Ecosystem diversity

Soils are remarkably complex and dynamic environments and hence typically comprise a wide range of habitat types for organisms over a range of dimensions from micrometre to the landscape scale (Berg, 2012; Foster, 1988; Oades & Waters, 1991). It is this highly heterogeneous nature of soil, particularly at the microhabitat level, that is responsible for its considerable biodiversity (Jeffery et al., 2010). Biological activity can take place in the soil pores, aggregates, detritus, rhizosphere and the drilosphere (Nielsen, 2019).

As described by Six et al. (2004), there are four different soil pore classifications, which are home to different microorganisms: (i) macropores (home to microarthropods); (ii) pores between macroaggregates (nematodes); (iii) pores between microaggregates, within macroaggregates (protists, small nematodes and fungi); and (iv) pores within microaggregates (bacteria).

## Functional diversity and ecosystem services

**Figure 3:**
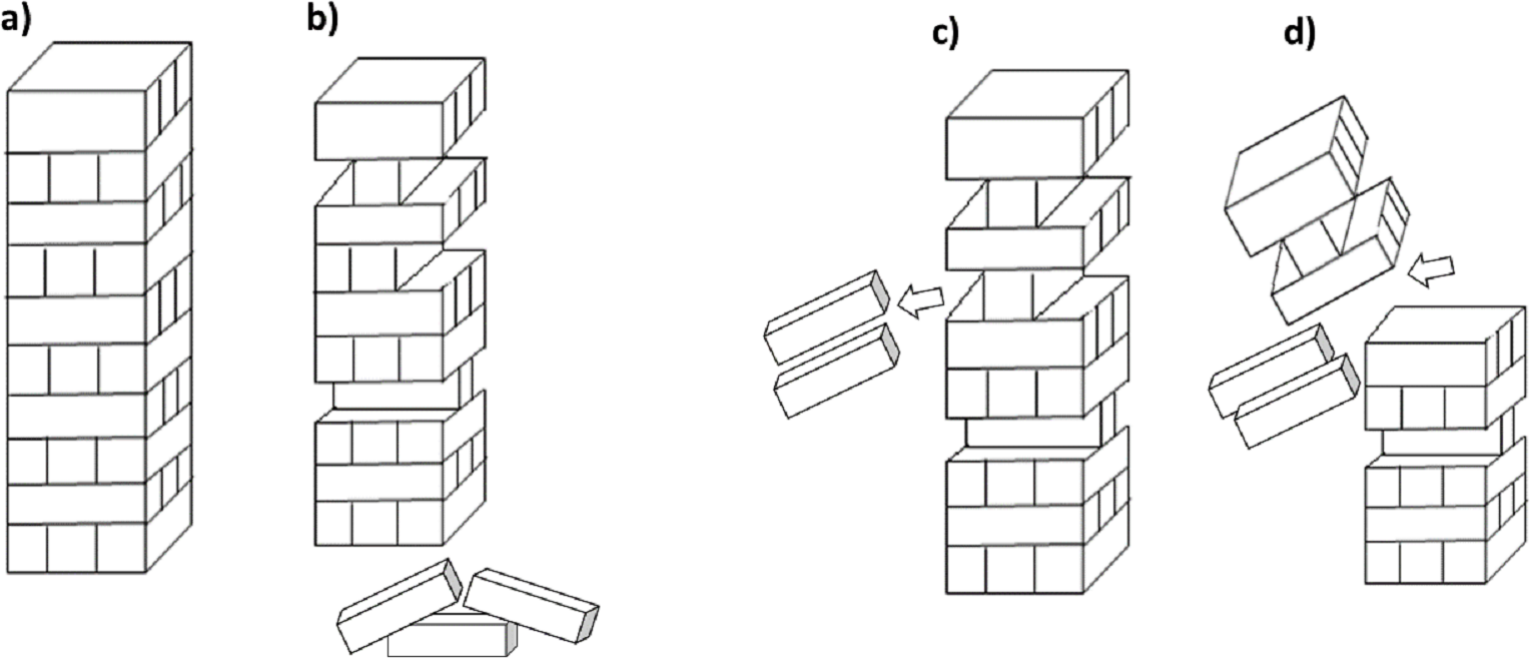
Jenga™ analogy for functional diversity. (A) A stable tower represents the function of the system and each of the blocks represent a species, a number of species in the system contribute to the function; (B) some species can be removed during a disturbance but the overall function of the system can still be maintained (i.e. some bricks can be removed without the tower falling) however (C) some species may have more importance in maintaining the function compared to others, or there may be a critical limit where too many species are removed which will lead to (D) collapse of the systems function.

Functional diversity considers the variety and number of taxa that undertake contrasting functional roles in the soil. Measuring diversity in this way allows an emphasis on the function of biodiversity within an ecosystem, rather than the specific species that comprise the diversity. Species diversity is thought to be important because it is synonymous with ecosystem health, leading to ecosystem supporting functions, and ultimately ecosystem services. However, there is often considerable functional redundancy in soil (Walker, 1992; Wellnitz & Poff, 2001), primarily due to its tremendous heterogeneity. Functional redundancy within a soil is where certain species contribute in equivalent ways to precise functions, such that one species may substitute for another (Fetzer et al., 2015; Louca et al., 2018). In effect this means the loss of taxonomic diversity may not necessarily lead to the loss of soil functions as more than one species may be performing the same task (e.g., decomposition of an organic compound), or have the same functional niche (Rosenfeld, 2016). The analogy of the game Jenga™ is often used in this instance (Fig. 3), where pieces can be removed and replaced, with the stability of the tower depending on the importance of the pieces that are being removed (De Ruiter et al., 2005).

A review of soil biodiversity relationships with C cycling by Nielsen et al. (2011) compiled studies where species richness was manipulated to observe changes in C dynamics. They reported that in low diversity systems, there was a positive relationship between C cycling and richness, with indications that community composition and loss of species with specialized functions in soil may have a greater effect on C dynamics (Nielsen et al., 2011). In addition, organisms may not necessarily occupy the same ecological niche. A loss of one of two species of equivalent function may be a form of functional debt where one species niche that is lost from a soil may be more competitive or have a wider niche, able to function when the second species may be uncompetitive or excluded from certain habitats within the soil. (e.g., one species may not survive flooding). In this way taxonomic diversity of species may remain significant. Functional debts can result from biodiversity loss for example a reduction in decomposition caused by simplification of food webs (Haddad et al., 2015). It is important to note that decomposition and C dynamics are only one of many functions and services performed by soil biota. The concept of functional redundancy may only consider the measure of a limited number of functions, thus overlooking specialized functions that are performed by select groups of organisms. Metagenomic analysis and further exploration into functional genes have the potential for providing insight into the relationship between taxonomic diversity and functional diversity.

Several reviews have outlined the importance of soil biodiversity and their contribution to ecosystem services in recent years for soil management (Barrios, 2007; Bender, Wagg & Van der Heijden, 2016; Briones, 2014; Nielsen, Wall & Six, 2015), the dynamics of soil food webs (Morriën, 2016), and the risk of extinction (Veresoglou, Halley & Rillig, 2015). They all demonstrate that the activities of the soil biota are essential to provide many of the ecosystem services that are considered typical of the wider landscape. These stretch much beyond supporting the production of food and fibre and extend into issues such as erosion control and pollution attenuation (Jónsson & Davídsdóttir, 2016). The primary functions of soil biota include (i) nutrient cycling; (ii) regulation of water flow and storage (iii) regulation of soil and sediment movement; (iv) biological regulation of other biota (including pests and diseases); (v) soil structural development and maintenance; (vi) the detoxification of xenobiotics and pollutants; and (vii) the regulation of atmospheric gases. These functions contribute to a range of ecosystem services such as: (1) provisioning of food, fibre and biotechnology, (2) regulating climate, atmospheric composition, and hydrological services, (3) supporting soil formation, habitat and biodiversity conservation and (4) maintaining cultural services as natural capital (Brussard, 2012). The ecosystem services concept provides an understandable and translatable outcome of the role of soil biodiversity in a manner that allows people to recognise its impacts on their lives.

Developing a generic framework to assess the functions and services provided by soils has been challenging, especially given the heterogeneity of soil and gaps in data (Tzilivakis, Lewis & Williamson, 2005). The complexity of the soil system means that we still understand relatively little about its biology. Only ca. 1.5% of soil microorganisms have been characterized (Baveye, 2009) compared to 80% of plants (Jeffery et al., 2010). Despite these challenges, great advances have been made in recent years concerning our knowledge of living biomass in the soil (Bardgett & Van Der Putten, 2014; Nielsen, Wall & Six, 2015; Wagg et al., 2014).

## Review of 2010 (expert defined) threats to soil biodiversity

In order to identify a complete register of the potential threats to soil biodiversity published in the journal literature a comprehensive search was completed on the Web of Science. The 46 papers returned by Web of Science can be found in Table 1, which includes details of the search terms. We were interested to compare how global scientific activity has related to the threats identified by the expert panel in 2010. Although the term biodiversity was explicitly stated in the search, the majority of the papers examined changes in abundance and diversity of individual groups of soil biota, rather than soil biodiversity as a whole, encompassing all levels of the soil food web.

**Figure 4:**
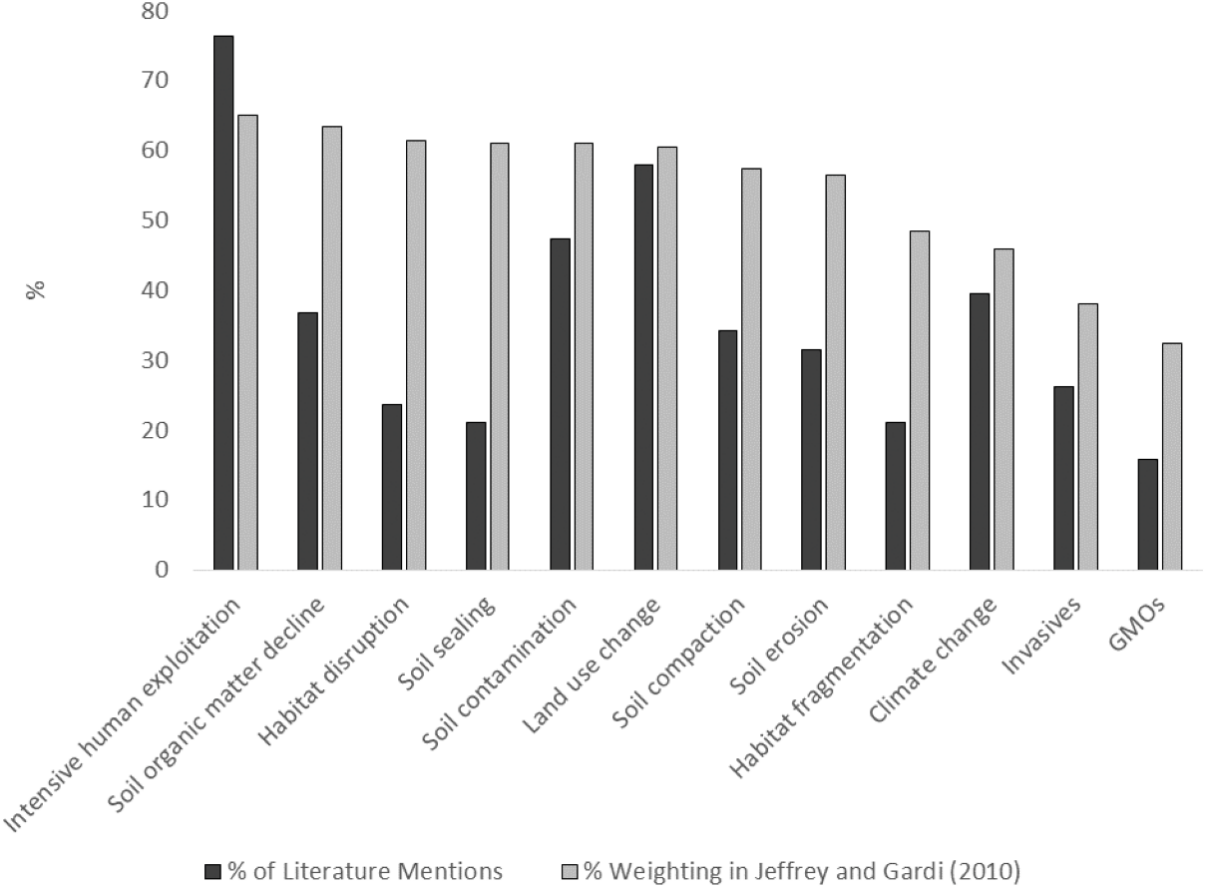
Findings of expert assessment of threats to soil biodiversity versus published literature on threats to soil biodiversity. Grey bars represent expert assessment assessment weight (%) in Jeffery & Gardi( 2010) black bars represent actual declarations in the journal literature.

The 12 identified threats to soil biodiversity, and the weightings given in the expert assessment in Jeffery & Gardi (2010) can be found in Fig. 4. We have also provided information on the proportion of the 46 references that state each of these potential threats in the text. The majority of focus in the literature on intensive human exploitation, and the lowest on GMOs which is in concordance with the expert assessment. However, this is where the similarities, in respect to order of priority, ends. Of the 12 threats to soil biodiversity provided in the expert assessment in Jeffery & Gardi (2010), the four that are most commonly specified in the literature are: (i) intensive human exploitation; (ii) land use change; (iii) soil contamination; and (iv) climate change.

**Table 1:** Threats identified in the literature. Threats declared in the 46 papers returned by Web of Science topic search threat* “soil biodiversity” on 27th November 2019. These are categorised (in rows) by whether they were either experimental (E), literature review (L) or meta-analytical (M) papers. The studies are categorised (in columns) into broad “threat” issues considered in each paper including Climate Change, Land Use Change, Intensive Human Exploitation, Decline in Soil Health or Plastic; followed by detailed listing of threats.

	Climate change		Land use change							Intensive human exploitation					Decline in soil health									Plastic	
**E**xperimental, mapping or workshop/ **L**iterature Review/ **M**eta-analysis	Climate change	Soil temp/moisture	Land use change	Deforestation	Conversion to ag	Soil sealing	Habitat fragmentation	Habitat disruption	Invasive species	Human exploitation	Intensive agriculture				Soil Degradation	Soil organic matter decline	Soil contamination	Soil compaction	Soil erosion	Soil salinization	Decline in soil nutrients	Atmospheric deposition (N and S)	Microplastics	Plastic mulch	Reference
											Intensive agriculture	Tillage	Chemicals	GMOs											
E													X					X							Meng et al. (2019)
E								X																	Spaans et al. (2019)
L	X	X	X	X		X		X	X		X		X				X					X	X		Geisen, Wall & van der Putten (2019)
M			X								X														Cameron et al. (2019)
E												X	X												Gu et al. (2019)
E			X			X				X					X	X	X	X	X	X					Tibbett et al. (2019)
E			X	X								X													Goss-Souza et al. (2019)
M			X	X	X				X			X	X					X							Franco et al. (2019)
E																						X			Shao et al. (2018)
L	X		X	X	X		X	X	X		X	X	X				X	X	X	X					Reinecke & Reinecke (2018)
E			X								X		X			X				X	X				Toth, Hornung & Baldi (2018)
M	X	X																	X						Hamidov et al. (2018)
E	X	X	X	X			X	X		X							X		X	X					Napierala, Ksiązkiewicz-Parulska & Błoszyk (2018)
E			X	X								X													Peerawat et al. (2018)
E																	X								Conti et al. (2018)
E		X									X							X						X	Schirmel et al. (2018)
E		X	X							X							X				X				Kostecka et al. (2018)
M									X																Ferlian et al. (2018)
L									X			X	X			X		X	X						Techen & Helming (2017)
E	X		X			X			X	X				X		X	X	X	X	X					Li et al. (2017)
E	X	X	X			X	X	X	X	X		X	X	X		X	X	X	X	X					Aksoy et al. (2017)
E			X									X	X												Castañeda & Barbosa (2017)
E			X								X											X			Zhang et al. (2017)
L	X	X									X		X			X	X								Pagano et al. (2017)
E	X					X					X					X	X	X	X	X					Griffiths et al. (2016)
E			X								X	X				X		X							Schorpp & Schrader (2016)
E	X		X			X	X			X			X	X		X	X	X	X	X					Orgiazzi, Bardgett & Barrios (2016)
L																	X								Agathokleous et al. (2016)
L	X		X			X		X								X	X	X	X						Montanarella & Panagos (2015)
E						X											X	X		X					Martin et al. (2015)
L	X	X	X								X	X				X			X						Acosta-Martínez et al. (2015)
E													X												Van Geel et al. (2015)
																									
E			X		X						X					X									Tsiafouli et al. (2015)
L													X												Chagnon et al. (2015)
E			X									X	X												Lavelle et al. (2014)
L											X	X	X												Clermont-Dauphin et al. (2014)
E	X		X			X	X	X	X	X	X			X	X	X	X	X	X						Gardi, Jeffery & Saltelli (2013)
L	X		X	X							X		X				X								Huang et al. (2013)
E	X		X																						Carpenter et al. (2012)
E													X												Jordaan, Reinecke & Reinecke (2012)
E			X				X	X																	Galanes & Thomlinson (2011)
E	X		X			X	X	X	X	X				X		X	X	X	X						Jeffery & Gardi (2010)
L	X		X				X	X	X	X	X			X	X	X	X	X	X						Gardi et al. (2009)
E	X							X	X	X	X						X								Wall et al. (2004)
L		X										X	X				X				X				Bongers & Bongers (1998)
L			X	X			X	X	X				X				X								Hagvar (1998)

However, it is important to note that there have been other potential threats to soil biodiversity that are not included in the Jeffery & Gardi (2010) expert assessment, such as soil salinization and the emerging threat of plastics. Many also elaborate, for example, what aspect of intensive human exploitation poses a threat to soil biodiversity, such as the use of chemicals in agriculture (Table 1). These emerging threats, and specifics, may influence mapping of threats to soil biodiversity in the future. In fact, the emergence of soil salinization as a threat to soil biodiversity may have led to its addition to the expert assessment and mapping in Orgiazzi et al. (2016).

## Identifying drivers and threats to soil biodiversity

The threats identified in the literature (Table 1) can be categorised into five broad themes, in order of the number of declarations in the 46 selected papers (Table 2): (i) *Intensive human exploitation*, including intensive agriculture using tillage, chemical fertilisers, pesticides, liming and GMOs; (ii) *Land use change*, including deforestation, conversion to agriculture, soil sealing and habitat fragmentation and disruption; (iii) Soil degradation resulting in a *decline in soil health/ quality*, such as organic matter decline, contamination, compaction, erosion, salinization and loss of available nutrients; (iv) *Climate change*, particularly the effects of a change in soil temperature and moisture; and (v) *Plastics*.

**Table 2:** Percentage of declarations within each threat category in the 46 papers returned by Web of Science topic search threat* “soil biodiversity” on 27th November 2019.

**Threat category**	**% Publications mentioning threat**
Intensive human exploitation	78
Land use change	72
Decline in soil health/ quality	70
Climate change	43
Plastics	4

With these five threat categories in mind, further searches in Web of Science were conducted, also on 27th November 2019, that focussed on each theme individually, regardless of whether they included the word ‘threat’ or not (Table 3). For example, “plastic*” & “soil biodiversity”. Climate change returned the most papers, followed by (in descending order): land use change; intensive human exploitation; soil health decline; and plastics (Table 3). The order shown in Table 3 differs from the number of declarations of each category in the selected papers from the threat* “*soil biodiversity*” listed in Table 2. In particular, a far greater number of papers were returned for *“climate change” & “soil biodiversity”.* However, with the removal of the word ‘threat’ from the search references are being returned that not only report the impacts of climate change on soil biodiversity, but also the ecosystem services provided by soil biodiversity in terms of climate change mitigation (e.g., Lubbers et al., 2019). From this broader bibliographic analysis, we concluded that threats to soil biodiversity was the appropriate focus of this review, and the use of the search term threat* “*soil biodiversity*” was therefore rational in this instance.

**Table 3:** Major soil threat terms used in Web of Science searches (27th November 2019) and the number of results that were returned by each search.

**Search term**	**Number of papers returned**
“climate change” & “soil biodiversity”	106
“land use change” & ”soil biodiversity”	41
“intensive human exploitation” OR “intensive agriculture” & ”soil biodiversity”	15
Decline in soil health & “soil biodiversity”	12
“Plastic” & “soil biodiversity”	12

These five categories cannot be considered in isolation as they interact and influence one another. For example, degradation of *soil quality* such as organic matter decline, compaction etc. is often a result of *land use change* from productive ecosystems to *intensive agriculture.* This makes quantifying soil threats very complicated.

Intensive human exploitation was the most commonly investigated threat category in the literature search threat* “soil biodiversity” (Table 2), most probably because it has knock-on effects on soil health which will impact soil biodiversity*.*

Particular consideration was given to agricultural intensification, with 86% of the papers stating intensive human exploitation as a threat referring to agricultural intensification. The pressures that cause agricultural intensification include population growth, food production disparities, urbanization, and a growing shortage of land suitable for agriculture (FAO, 2017). Agricultural intensification has been shown to have a marked effect on soil biodiversity across Europe (Tsiafouli et al., 2015). The agricultural techniques/management that can lead to loss of soil biodiversity are monoculture cropping, removal of residues, soil erosion, soil compaction (both due to degradation of the soil structure) and repeated application of chemicals (Nielsen, Wall & Six, 2015; Wachira et al., 2014). Discussion of general use of chemicals, such as fertilizers and pesticides, account for the largest proportion, of papers examined during this review, discussing agricultural intensification as a threat to soil biodiversity (60%). The use of chemicals, as part of agricultural intensification, are a significant cause of soil biodiversity loss, although the reported effects are not always consistent. Pesticides can deplete or disrupt non-target invertebrate (e.g., earthworms) and soil microbial communities, and associated functions such as nitrogen fixation and nutrient uptake (Chagnon et al., 2015; Jordaan, Reinecke & Reinecke, 2012; Mahmood et al., 2016; Pagano et al., 2017). However, as a result of potential functional redundancy, it is uncertain to what degree soil communities can absorb the effects of pesticides before ecosystem services are impacted (Chagnon et al., 2015). Artificial liming has also been found to impact on soil biodiversity as result of modification of soil pH. For example, bacteria abundance and diversity was found to increase with increasing pH, as a result of liming in arable soil (Rousk et al., 2010). A pH induced change in the microbial community will then have a knock-on effect on whether bacterial or fungal feeding nematodes dominate in a soil (Bongers & Bongers, 1998).

Intensive soil management activities that disrupt the structure of the soil with heavy machinery causing compaction, i.e., by increasing bulk density, reducing habitable pore space, can influence the diversity of environments in which different microorganisms can thrive (Hattori, Hattori & McLaren, 1976; Janušauskaite, Kadžiene & Auškalniene, 2013). Compaction of soil can also impact on larger burrowing fauna as burrowing compacted soils requires more energy, at the expense of other activities such as foraging, mating etc. (Martin et al., 2015). In addition, an increase in bulk density is often associated with a decrease in organic matter and soil moisture content, which can also influence community assemblages (Nielsen et al., 2014).

Mechanical tillage, combined with agricultural lands being bare for long periods after harvest, has been shown to increase instances of soil erosion by wind and water. Tillage can also risk injury to larger soil fauna and anecic earthworms, for example (Schorpp & Schrader, 2016).

Incorporation of organic residues into the subsoil during tillage will also influence soil biodiversity. This is largely a result of varying metabolisms between organisms; for example, input of fresh decomposable organic residues has a stronger effect on bacterial communities because they are able to metabolise labile compounds quickly, therefore dominating over other organisms (He et al., 2016; Marschner, Umar & Baumann, 2011). Enrichment opportunist nematodes, and endogeic earthworms will also thrive in systems with fresh organic matter incorporation into a plough layer during tillage (Bongers & Bongers, 1998; Schorpp & Schrader, 2016).

### Decline in soil health

Intensive human exploitation of soil often leads to subsequent deterioration in soil health such as decline in organic matter, soil compaction, soil contamination and soil salinization, resulting in a decline in soil biodiversity (Aksoy et al., 2017).

Decline in soil organic matter has been a general feature of tillage agriculture (Janzen, 2006) and C losses have been found to be occurring at a national scale in the UK, for example (Bellamy et al., 2005). As the source of energy underpinning food-webs, C losses from organic matter leads to reduced biodiversity (Aksoy et al., 2017).

Soil contamination can come from a variety of sources including agriculture, industry, waste management and transport, and thus contamination can occur in both rural and urban soils. Soil contamination has been shown to be detrimental to the soil community on several occasions whether it be heavy metals (Huang et al., 2013), organic pollutants, or atmospheric deposition of nitrogen (Shao et al., 2018).

Soil salinization has emerged as a threat to soil biodiversity. Soil salinization, as a result of over-abstraction of ground water to meet demands of population growth, urbanisation, agriculture and industry, and subsequent seawater intrusion, excess use of fertilizers and municipal wastewater can lead to structural collapse of soil aggregates, reduction in organic matter and soil erosion, which will negatively impact soil biodiversity (Daliakopoulos et al., 2016). Water availability is significantly lower in saline soils, which will further compound negative effects on soil biodiversity (Martin et al., 2015)

### Land use change

Globally, more than half of native forest land cover has been converted to agricultural land (Huang et al., 2013) and an estimated 2.3 million km^2^ of forest was lost between 2000-2012, the majority of which occurred in the tropics (Hansen et al., 2013). Deforestation in tropical forests has been shown to negatively impact soil microbes, mesofauna and macrofauna (Franco et al., 2019; Peerawat et al., 2018). Land use change that results in less diverse vegetation assemblages such as conversion of semi-natural ecosystems to monocrop agriculture will negatively impact soil biodiversity (Figuerola et al., 2015). Well established semi-natural woodlands with a lot of deadwood, for example, are rich in microhabitat types and therefore increase ecosystem diversity (Napierala, Ksiązkiewicz-Parulska & Błoszyk, 2018). Destruction of habitat, therefore, will favour species that have a wide range of habitats and the ability to adapt to new conditions, outcompeting those that can only survive within a narrow range of conditions (Kostecka et al., 2018).

Land conversion for agricultural activities is not the only threat posed to soil biodiversity. Population growth, and subsequent urbanization of green spaces, has led to increased soil sealing. An estimated 275 ha of soil sealing per day has been reported in EU countries, decreasing soil biodiversity by blocking organic matter inputs and water infiltration (Montanarella & Panagos, 2015). Soil sealing in one area of a river catchment may also lead to increased flooding and soil erosion elsewhere in the catchment causing additional, indirect, threats to soil biodiversity.

### Climate change

Climate change can impact soil biodiversity both directly and indirectly. Climate change can alter the temperature and moisture regime of the soil thereby impacting soil biodiversity (Aksoy et al., 2017; Hamidov et al., 2018). Climate change can also lead to extreme weather events including heavy rainfall and drought, along with sea level rise. Therefore, climate change can indirectly threaten soil biodiversity through increased soil erosion and salinization, for example (Daliakopoulos et al., 2016; Hamidov et al., 2018). Climate change has also led to cultivation of soils where agriculture was previously not possible, with northward shifts of agricultural lands (King et al., 2018), including melting permafrost soils (Hamidov et al., 2018). Furthermore, proposed mitigation strategies for greenhouse gas emissions, such as amendment of soil with biochar could have an impact on soil biodiversity. Biochar, as a source of C has shown to benefit soil microorganisms, however, the effects on macroorganisms has not been examined to the same degree and contaminant compounds in biochar have proven to impact survival and reproduction of Collembola (Conti et al., 2018; Lehmann et al., 2011).

### Plastics

Global production of plastics has increased significantly in the last 60 years rising from 1.7 million t in 1950 to 299 million t in 2013 (Duis & Coors, 2016). Despite only a small volume of literature to date on the threats of plastic to soil biodiversity, we believe this is a potential emerging threat. Plastics can influence soil biodiversity in several ways both directly and indirectly. Plasticulture in agriculture, using a polyethene film as a mulch layer on the soil surface has been shown to reduce diversity soil invertebrates and soil microbial activity as a result of change in temperature and moisture regimes (Schirmel et al., 2018) and shift the microbial community towards thermophilic, anaerobic and detritivorus species (Steinmetz et al., 2016). Moreover, plastic mulching may lead to increased runoff of pesticides to the wider catchment area, adversely affecting soil biodiversity in the wider environment (Steinmetz et al., 2016). Plastics can also act as a soil contaminant in their own right, particularly small (>5 mm) fragments of plastic, known as microplastics. Microplastics can occur as a product of decomposition of larger plastic waste (Cao et al., 2017), including plastic mulching and polytunnels in agricultural land (Horton et al., 2017), or from products such as exfoliating facial cleansers (‘microbeads’), toothpastes, cleaning agents and textile fibres entering wastewater and incorporated in sewage sludge which can be applied to agricultural land (Bouwmeester, Hollman & Peters, 2015; De Souza Machado et al., 2018). Additives of plastic products (plasticisers, retardants, antioxidants etc.) and adsorbed chemicals could also pose a potential threat to soil biodiversity as contamination (Bouwmeester, Hollman & Peters, 2015; Chae & An, 2018).

Plastic pollution of the terrestrial and aquatic environment is currently receiving worldwide attention in the media and research (Chae & An, 2018), with aquatic systems receiving much of the attention to date (Cao et al., 2017). However, soils are likely to be a substantial sink for microplastic (Hurley & Nizzetto, 2018). In fact, agricultural soils alone might store more microplastics than oceanic basins (Nizzetto & Futter, 2016), with the effects on soil biota widely unknown. Therefore, microplastics could be considered as an emerging threat to soil biodiversity that is receiving increased research attention, and needs continued research in the future (Rillig & Bonkowski, 2018). Particularly as earthworms have been demonstrated to transport microplastics through the profile, potentially exposing other organisms in the subsoil to this new potential threat (Rillig, Ziersch & Hempel, 2017). The presence of microplastics in soil can also affect soil structure negatively, reducing the stability of soil aggregates (Lehmann, Fitschen & Rillig, 2019), which may in turn lead to negative consequences for soil biodiversity. Recently it has also been postulated that microplastics may have evolutionary implications for the soil biota generally which, if so, may lead to unpredictable outcomes for the biodiversity in soil (Rillig et al., 2019).

The majority of studies to date on the negative effects of microplastics on soil biology has focussed on earthworms (Huerta Lwanga et al., 2016) and springtails (Kim & An, 2019; Maaßet al., 2017). For example, Huerta Lwanga et al. (2016) showed that growth rate and mortality of *Lumbricus terrestris* is influenced by ingestion of microplastics at concentrations of 5% v/v and above. This microplastic concentration may seem high, however, concentrations of microplastic were found to be as high as 6.7% in some industrial areas of Sydney, Australia (Fuller & Gautam, 2016). Therefore, further research on the effects of plastic pollution on soil dwelling invertebrates, insects, and microorganisms need to be urgently considered if plastics are to be seriously considered as a threat to soil biodiversity (Chae & An, 2018).

## Conclusions

Threats to soil biodiversity are many and varied, and the published literature has only just begun to unravel the complexity of soil biological systems. We barely know what is there, let alone their breadth of functional roles, niche partitioning and interaction between these organisms.

Many biodiversity studies do not focus on biodiversity in its strictest sense, rather these studies examined either changes in abundance and/or diversity of individual groups of soil biota, rather than soil biodiversity as a whole, encompassing all levels of the soil food web.

Published global scientific activity was only partially related to the threats identified by the expert panel in 2010. The number of threats and the priority given to the threats (by number of publications) were quite different, indicating a disparity between research actions versus perceived threats. The lack of research effort in key areas of high priority in the threats to soil biodiversity are a concerning finding and requires some consideration and debate in the research community.

## Additional Information and Declarations

### Data Availability

The following information was supplied regarding data availability: This is a literature review article and did not generate raw data.
